# Magnetic resonance-derived hepatic uptake index improves the identification of patients at risk of severe post-hepatectomy liver failure

**DOI:** 10.1093/bjs/znaf103

**Published:** 2025-05-29

**Authors:** Wolf C Bartholomä, Stefan Gilg, Peter Lundberg, Peter N Larsen, Ville Sallinen, Malin Sternby Eilard, Jozef Urdzik, Gert Lindell, Torkel B Brismar, Eva Fallentin, Ali Ovissi, Andreas Socratous, Tomas Bjerner, Sophie Kollbeck, Jens Tellman, Fredrik Holmquist, Nils Dahlström, Mischa Woisetschläger, Bergthor Björnsson, Ernesto Sparrelid, Per Sandström

**Affiliations:** Department of Radiology and Department of Health, Medicine, and Caring Sciences, Linköping University, Linköping, Sweden; Centre for Medical Image Science and Visualization (CMIV), Linköping University, Linköping, Sweden; Division of Surgery and Oncology, Department of Clinical Science, Intervention, and Technology, Karolinska Institutet, Karolinska University Hospital, Stockholm, Sweden; Department of Radiology and Department of Health, Medicine, and Caring Sciences, Linköping University, Linköping, Sweden; Centre for Medical Image Science and Visualization (CMIV), Linköping University, Linköping, Sweden; Department of Radiation Physics and Department of Health, Medicine, and Caring Sciences, Linköping University, Linköping, Sweden; Department of Surgical Gastroenterology and Transplantation, Rigshospitalet, Copenhagen University Hospital, Copenhagen, Denmark; Department of Gastroenterological Surgery, Helsinki University Hospital and University of Helsinki, Helsinki, Finland; Transplant Institute, Institute of Clinical Sciences, Sahlgrenska Academy at University of Gothenburg, Sahlgrenska University Hospital, Gothenburg, Sweden; Department of Surgical Sciences, Uppsala University, Uppsala, Sweden; Department of Surgery, Skåne University Hospital Comprehensive Cancer Centre, Lund University, Lund, Sweden; Department of Radiology, Karolinska University Hospital in Huddinge, Stockholm, Sweden; Department of Radiology, Rigshospitalet, Copenhagen University Hospital, Copenhagen, Denmark; Department of Radiology, University of Helsinki and Helsinki University Hospital, Helsinki, Finland; Department of Radiology, Sahlgrenska University Hospital, Gothenburg, Sweden; Department of Radiology and Department of Health, Medicine, and Caring Sciences, Linköping University, Linköping, Sweden; Centre for Medical Image Science and Visualization (CMIV), Linköping University, Linköping, Sweden; Department of Surgical Gastroenterology and Transplantation, Rigshospitalet, Copenhagen University Hospital, Copenhagen, Denmark; Department of Radiation Physics and Department of Health, Medicine, and Caring Sciences, Linköping University, Linköping, Sweden; Department of Medical Imaging and Physiology, Skåne University Hospital, Lund University, Lund, Sweden; Department of Radiology and Department of Health, Medicine, and Caring Sciences, Linköping University, Linköping, Sweden; Centre for Medical Image Science and Visualization (CMIV), Linköping University, Linköping, Sweden; Department of Radiology and Department of Health, Medicine, and Caring Sciences, Linköping University, Linköping, Sweden; Centre for Medical Image Science and Visualization (CMIV), Linköping University, Linköping, Sweden; Department of Surgery and Department of Biomedical and Clinical Sciences, Linköping University, Linköping, Sweden; Division of Surgery and Oncology, Department of Clinical Science, Intervention, and Technology, Karolinska Institutet, Karolinska University Hospital, Stockholm, Sweden; Department of Surgery and Department of Biomedical and Clinical Sciences, Linköping University, Linköping, Sweden

## Abstract

**Background:**

Post-hepatectomy liver failure (PHLF) is a leading cause of mortality after major liver resection. Accurate preoperative risk assessment is essential, yet current methods have limitations. Gadoxetic acid-enhanced MRI (Gd-EOB MRI) enables both morphological and functional evaluation of the liver. The aim of this study was to evaluate the efficacy of the hepatic uptake index (HUI) obtained from routine preoperative Gd-EOB MRI for identifying patients at risk of severe PHLF.

**Methods:**

This observational retrospective multicentre study included 292 patients who underwent major hepatectomy between 2010 and 2020 in Sweden, Denmark, and Finland. Preoperative Gd-EOB MRI was performed for each patient and the HUI, hepatic uptake index of the standardized future liver remnant (sFLR-HUI), and Model for End-Stage Liver Disease Version 3 (MELD 3) score were evaluated. Statistical analyses included logistic regression and receiver operating characteristic (ROC) curve assessment to determine cut-off values and discriminative accuracies for severe PHLF (International Study Group of Liver Surgery grades B and C).

**Results:**

Among the 292 patients, 25 (8.6%) developed severe PHLF. Patients with severe PHLF had significantly lower HUI and sFLR-HUI values (*P* < 0.001). The HUI demonstrated superior discriminative performance for severe PHLF (area under the curve (AUC) 0.758) compared with volume-only assessments, such as the standardized future liver remnant (sFLR) (AUC 0.628). Combining the HUI with the MELD 3 score improved performance further (AUC 0.803).

**Conclusion:**

The HUI obtained from routine Gd-EOB MRI outperforms volume-based biomarkers (sFLR) for identification of patients at risk of severe PHLF. Incorporating image-derived functional assessments, such as the HUI, with independent biomarkers, such as the MELD 3 score, may optimize preoperative risk stratification for severe PHLF and improve outcomes after major hepatectomy.

## Teaser

MRI-based surrogate markers, such as the hepatic uptake index (HUI), can effectively predict severe post-hepatectomy liver failure (PHLF) and surpass risk stratification based on volume alone. Combining an MRI-based marker with a clinical score, such as the Model for End-Stage Liver Disease Version 3 (MELD 3) score, further increases performance. The findings suggest that integrating MRI-based regional functional data into existing or newly created multivariable models is the most promising approach for the prediction of severe PHLF.

## Introduction

Major liver resection is a standard treatment for malignant liver tumours suitable for surgery. Although a significant proportion of the liver can regenerate after major resection, allowing survival even after 70–80% of the healthy liver volume has been removed^1^, post-hepatectomy liver failure (PHLF) remains the leading cause of mortality after major liver surgery, with an incidence of 5–9%^2,3^.

Safe resection without increasing the risk of liver failure is challenging and no uniform definition of PHLF exists. The International Study Group of Liver Surgery (ISGLS) criteria^4^ are commonly used, allowing a graded assessment of PHLF compared with other dichotomizing criteria, such as the 50/50 criteria of Balzan *et al*.^5^ and peak bilirubin levels^6^. However, the ISGLS criteria depend on laboratory values from the fifth postoperative day, limiting their use in clinical settings^7^. Preliminary findings suggest that early postoperative laboratory data may be useful, but require further validation^8^.

As there is no effective treatment for PHLF, better preoperative stratification methods are needed. Preoperative volumetry of the future liver remnant (FLR), through CT or formulae based on body surface area (BSA), is used to reduce PHLF risk, ensuring that the FLR exceeds 20% of the total functional liver volume in patients with healthy liver parenchyma, 30% in those with steatosis, and 40% in those with cirrhosis^1,9–11^.

Functional scores, such as the aspartate aminotransferase-to-platelet ratio index (APRI) and the albumin-bilirubin (ALBI) score, can indicate increased risks of severe PHLF and mortality after major hepatectomy^12^, especially when integrated in a multivariable model, but liver volume and local FLR function are not considered^13^.

Indocyanine green (ICG) clearance^14,15^ and the liver function maximum capacity (LiMAx) test^16^ assess preoperative liver function, but are expensive, not widely available, and only measure global liver function.

Hepatobiliary scintigraphy (HBS) with ^99m^Tc-mebrofenin shows potential^17^, although a multicentre study by Olthof *et al*.^18^ revealed that the predictive value of HBS was not significantly superior to remnant liver volume alone.

MRI with hepatocyte-specific contrast agents, such as gadoxetic acid (Gd-EOB), is a standard examination in patients scheduled for major liver resection. It can assess preoperative liver function both globally and segmentally, including the FLR^19^. It can also be used for volumetry. Hence, gadoxetic acid-enhanced MRI (Gd-EOB MRI) could serve as a valuable one-stop tool for preoperative risk assessment.

Different Gd-EOB MRI approaches exist to estimate liver function, from relatively simple approaches adjusting liver signal intensity with splenic intensity to sophisticated dynamic function models^20^. One simple, but effective, approach is the hepatic uptake index (HUI). It can be derived from routine MRI examinations and correlates well with established liver function tests, such as ICG clearance^21^.

Several researchers have studied the value of Gd-EOB MRI for preoperative PHLF risk stratification^19,22^, but these studies were often limited by small patient cohorts, particularly cohorts of patients with severe PHLF, or were single-centre studies using only one MRI scanner, limiting generalizability.

The aim of this observational, retrospective, multicentre study was to address these limitations by analysing one of the largest patient cohorts to date and evaluating whether routine preoperative hepatocyte-specific enhanced MRI, performed at various sites with different scanners and protocols, can improve the identification of patients at risk of severe PHLF.

## Methods

### Study design

This observational, retrospective, multicentre study was conducted at tertiary centres in Sweden, Denmark, and Finland. The requirement for informed consent was waived by each centre’s review board. The study was registered on clinicaltrials.gov (NCT04692259). Participating hospitals included: Linköping University Hospital, Karolinska University Hospital, Sahlgrenska University Hospital, Uppsala University Hospital, and Skåne University Hospital in Sweden; Rigshospitalet in Denmark; and Helsinki University Central Hospital in Finland.

### Study eligibility criteria

Patients who underwent preoperative Gd-EOB MRI before major hepatectomy between 1 January 2010 and 31 December 2020 were included. MRI examinations were performed within 8 weeks before surgery for patients without preoperative volume augmentation and within 2 weeks for those who underwent augmentation. Major hepatectomy was defined as resection of three or more Couinaud segments or more than one segment in patients with cirrhosis.

**Fig. 1:**
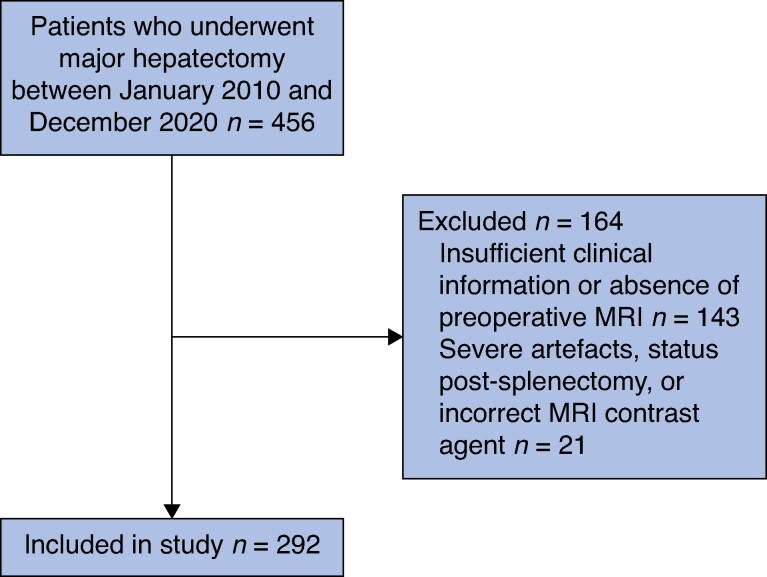
Flow chart of included and excluded study patients Flow chart showing patient inclusion for a study on major hepatectomy from January 2010 to December 2020. Out of 456 patients, 164 were excluded (143 due to insufficient clinical data or lack of preoperative MRI and 21 due to severe artefacts, prior splenectomy, or incorrect MRI contrast agent). The final study cohort included 292 patients.

Patients lacking necessary clinical information, aged <18 years, with excessive Gd-EOB MRI artefacts, or who had previously undergone splenectomy were excluded (*Fig. 1*).

### Calculation of total functional liver volume

The total functional liver volume was estimated using the standard liver volume formula-based BSA:


eTLV=−794+1267×BSA

where eTLV is the estimated total liver volume.

The BSA was calculated using Mosteller’s formula:


$$BSA=\;\sqrt{\frac{\left[height(cm)\times weight\;(kg)\right]}{3600}}$$

This eTLV was also used to calculate the standardized future liver remnant (sFLR), following Ribero *et al*.^23^:


$$sFLR=\;\frac{FLRV}{eTLV}=\frac{FLR}{-794+1267\times BSA}$$

where FLRV is the future liver remnant volume.

### Image acquisition techniques

Preoperative imaging of the liver and spleen was performed in the hepatobiliary phase, 20–30 min after the administration of 0.025 mmol/kg Gd-EOB. Routine clinical protocols were followed in each hospital. Scanners from Siemens (Erlangen, Germany), Philips (Amsterdam, The Netherlands), and General Electric (Boston, MA, USA) were used, including both 1.5 T and 3 T systems. T1-weighted fat-saturated volumetric sequences were acquired during the hepatobiliary phase and used for liver volumetry and signal intensity measurements.

### Determination of FLRV

A senior radiologist with 18 years of expertise in diagnostic MRI and a supervised fourth-year medical student segmented the FLR using a semi-automatic process involving MATLAB R2022b (MathWorks, Natick, MA, USA), Mialab (Novamia AB, Tullinge, Sweden), and SmartPaint version 1.5.1 software (free software, Filip Malmberg, Uppsala University); Mialab generated the initial segmentation, which was refined in MATLAB and then inspected and adjusted using SmartPaint. The radiologist and medical student were blinded to patient PHLF status.

### Measurement of liver and splenic signal intensities and calculation of the HUI

The same radiologist and student measured the signal intensity of each liver segment, as well as the spleen (Regions of interest (ROIs) with diameters ranging from 3 to 10 mm), avoiding vessels, focal lesions, and artefacts.

The HUI was calculated using the following formula:


$$HUI_{FLR}=FLRV\times \left[\frac{L_{HBP}}{S_{HBP}}-1\right]$$

where FLRV is in ml, *L_HBP_* is the mean signal intensity of the liver segments constituting the FLR, and *S_HBP_* is the mean signal intensity of the spleen in the hepatobiliary phase.

The hepatic uptake index of the standardized future liver remnant (sFLR-HUI) was calculated as follows:


$$HUI_{sFLR}=sFLR\times \left[\frac{L_{HBP}}{S_{HBP}}-1\right]$$

For simplicity, HUI and sFLR-HUI are used throughout.

### Calculation of the functional liver imaging score (FLIS)

For a randomly selected subcohort of the study cohort (155 patients; 142 with mild or no PHLF (91.6%) and 13 (8.4%) with severe PHLF) the FLIS was calculated. In accordance with previous studies^24,25^, the score was determined based on axial and coronal T1 images in the hepatobiliary phase as the sum of enhancement quality (comparison of signal intensity of the liver compared with the right kidney, 0–2 points), portal vein sign quality (comparison of the liver compared with the portal vein, 0–2 points), and excretion quality (assessment of contrast opacification of the biliary tree, 0–2 points).

### Laboratory data and clinical scores

Preoperative and postoperative laboratory data included total bilirubin, albumin, creatinine, platelet count, sodium level, prothrombin time, and international normalized ratio (INR). The sFLR, Model for End-Stage Liver Disease Version 3 (MELD 3) score, Charlson co-morbidity index, and ALBI score were calculated and recorded.

Resections were classified as right hemihepatectomy, extended right hemihepatectomy, left hemihepatectomy, extended left hemihepatectomy, and atypical resection. Atypical resections included cases in which multiple localized surgical resections were performed. Hemihepatectomies with local ablation were generally classified as hemihepatectomy.

### Definition of PHLF

PHLF is defined as the deterioration of liver function after surgery. According to the ISGLS, PHLF is characterized by an elevated INR and hyperbilirubinaemia on or after the fifth postoperative day^4^. PHLF severity is graded into three categories: grade A, where laboratory abnormalities are present, but do not require changes in clinical management; grade B, which necessitates altered clinical management without invasive treatment; and Grade C, which involves altered clinical management with invasive treatment. In this study, patients were either classified as ‘no or mild PHLF’ (comprising those with no PHLF and grade A PHLF) or ‘severe PHLF’ (grade B PHLF and grade C PHLF).

### Statistical analysis

SPSS^®^ (IBM Corp., Armonk, NY, USA) was used for statistical evaluation.

Patients without PHLF and those with mild PHLF (grade A) were grouped and compared with patients with severe PHLF (grades B and C).

Normality was assessed for the HUI, sFLR-HUI, FLR signal intensity, FLRV, MELD 3 score, ALBI score, and sFLR via the Kolmogorov–Smirnov test. Owing to nonnormal distributions, non-parametric tests were used for further analysis.

The Mann–Whitney *U* test was used for univariate analysis of significant differences. Spearman’s ρ was used to assess correlations between continuous variables, while Phi coefficients and Cramér’s V were used to evaluate associations between categorical variables. For correlations, 95% confidence intervals were calculated using bootstrapping (1000 samples) with bias-corrected and accelerated intervals.

Receiver operating characteristic (ROC) analysis determined the discriminative accuracy of the HUI, sFLR-HUI, MELD 3 score, ALBI score, FLRV, and sFLR for severe PHLF, with area under the curve (AUC) values recorded. Cut-off values were derived from Youden’s index and ROC coordinates. ROC analysis was also performed to assess the impact of bile duct resection and to compare the performance of the HUI with that of the FLIS in a random subset of patients for whom the FLIS was determined.

Binary logistic regression was conducted to calculate ORs. Z-scores improved comparability of ORs. Ninety-five percent confidence intervals were calculated using bootstrapping (1000 samples) with bias-corrected and accelerated intervals. Each variable was assessed for significance and those that significantly contributed to the prediction of PHLF (HUI, sFLR-HUI, FLR, and MELD 3 score) were then evaluated together in logistic regression. The predicted probabilities from this multivariable analysis were used in ROC analysis to assess specificity and sensitivity, and to determine cut-off values. ROC curves were compared for significant differences using pairwise comparison.

### Language editing

An artificial intelligence large language model (ChatGPT 4o) and external language editing services were used for language editing.

## Results

### Patient characteristics and group differences

Among the 292 patients studied, 25 (8.6%) developed severe PHLF. In patients with cirrhosis, severe PHLF was more prevalent (18.2% *versus* 3.0%), yielding a significant difference in background liver disease (*P* = 0.008). There were significant differences in the type of resection (*P* = 0.007); extended right hemihepatectomy was more common among patients with severe PHLF (34.8% *versus* 11.4%; adjusted residuals (AR) 3.2 for severe PHLF), whereas left hemihepatectomy was less common (0% *versus* 17.9%, AR 2.2 for no PHLF). Severe PHLF was also more common in patients who underwent bile duct resection (*P* = 0.008). See *Table 1*.

**Table 1:** Patient characteristics, laboratory values, measured biomarkers, and clinical scores, including statistically significant differences between patients without or with mild PHLF compared with patients with severe PHLF, as well as significant correlations; total study population, *n*  **=**  **292**

	No PHLF or mild PHLF (grade A)	Severe PHLF (grades B + C)	*P*	*R* (95% c.i.)
Patients per group	267 (91.4)	25 (8.6)		
**Patient characteristics**				
Age at surgery (years), median (i.q.r.)	64 (56–72)	65 (56–72)	0.738	
Sex, *n*			0.123	
Male	161	19		
Female	106	6		
ECOG performance status score, median (i.q.r.)††	0 (0)	0 (0–1)	0.085	
ASA grade, median (i.q.r.)‡‡	II (II–III)	II (II–III)	0.118	
BMI (kg/m^2^), median (i.q.r.)	25.4 (22.9–28.7)	26.3 (24.4–27.5)	0.860	
Diabetes mellitus	35 (13.1)	4 (16.0)	0.757	
CCI, median (i.q.r.)§§	8 (6–10)	8 (6–10)	0.879	
**Background liver disease**¶¶			0.008*†	0.230§ (0.071,0.494)
Normal	75 (31.8)	6 (27.3)		
Steatosis	43 (18.2)	6 (27.3)		
Fibrosis grade 1–3	96 (40.7)	5 (22.7)		
Cirrhosis	7 (3.0)	4 (18.2)		
Other	15 (5.6)	1 (4.5)		
**Type of resected tumour (histology)**			0.213†	
Colorectal metastases	161(60.3)	11 (44.0)		
Hepatocellular cancer	16 (6.0)	0 (0.0)		
Biliary cancer (intrahepatic, perihilar, extrahepatic)	39 (14.6)	6 (24.0)		
Gallbladder cancer	15 (5.6)	2 (8.0)		
Other (benign, other malign, not specified)	36 (13.5)	6 (24.0)		
**Type of resection**##			0.007*†	0.223§ (0.114,0.435)
Right hemihepatectomy	154 (58.6)	11 (47.8)		
Extended right hemihepatectomy	30 (11.4)	8 (34.8)		
Left hemihepatectomy	47 (17.9)	0 (0.0)		
Extended left hemihepatectomy	11 (4.2)	2 (8.7)		
Atypical resection	23 (8.7)	2 (8.7)		
**Bile duct resection**			0.016*‡	0.156¶# (0.003,0.309)
Resection of any type + bile duct resection	27 (10.1)	7 (28.0)		
**Preoperative volume augmentation**			0.215‡	
Preoperative augmentation of any type	16 (6.0)	3 (12.0)		
**Parameters**				
HUI, median (i.q.r.)	536 (351–944)	331 (189–441)	<0.001*	−0.250** (−0.345,−0.135)
sFLR-HUI, median (i.q.r.)***	34.0 (20.8–56.5)	18.1 (10.9–29.4)	<0.001*	−0.242** (−0.332,−0.142)
MELD 3 score, median (i.q.r.)†††	6 (6–8)	7.5 (7–11)	<0.001*	0.223** (0.096,0.352)
ALBI score, median (i.q.r)‡‡‡	−2.46 (−2.71 to −2.26)	−2.38 (−2.62 to −1.95)	0.107	
FLRV (ml), median (i.q.r.)	680 (523–976)	590 (456–787)	0.063	
sFLR (%), median (i.q.r.)***	41.1 (32.3–58.8)	36.8 (26.5–46.2)	0.037*	−0.124# (−0.234,0.000)
FLIS, median (i.q.r.)§§§	6 (6–6)	6 (4.5–6)	0.031*	−0.174 (−0.399,0.061)
**sFLR thresholds**				
sFLR >20% (patients above the threshold)***	254 (97.7)	22 (91.7)	0.088†	
sFLR >30% (patients above the threshold)***	210 (80.8)	15 (62.5)	0.035*†	0.125¶# (−0.011,0.266)
sFLR >40% (patients above the threshold)***	139 (53.5)	7 (29.2)	0.023*†	0.135¶# (0.015,0.255)

Values are *n* (%) unless otherwise indicated. *Statistically significant. *P* was calculated using the Mann–Whitney *U* test unless otherwise indicated: †chi-squared test and ‡Fisher’s exact test due to low counts. *R* was calculated using Spearman’s ρ unless otherwise indicated: §Cramér’s V and ¶Phi. Correlations are only reported for variables with statistically significant differences. #Correlation at the 0.050 level. **Correlation at the 0.001 level. ††Data missing for 54 patients (50 patients with no or mild PHLF and 4 patients with severe PHLF). ‡‡Data missing for two patients (both with no or mild PHLF). §§Data missing for 66 patients (62 patients with no or mild PHLF and 4 patients with severe PHLF); information was missing on the severity of liver disease in these patients. ¶¶Data missing for 34 patients (31 patients without or with mild PHLF and 3 patients with severe PHLF). ##Data missing for four patients (2 patients without or with mild PHLF and 2 patients with severe PHLF). ***Data missing for eight patients (7 patients with no or mild PHLF and 1 patient with severe PHLF). †††Data missing for four patients (3 patients with no or mild PHLF and 1 patient with severe PHLF). ‡‡‡Data missing for 116 patients (110 patients with no or mild PHLF and 6 patients with severe PHLF) . §§§Subcohort of 155 patients. Data missing for 147 patients (135 patients with no or mild PHLF and 12 patients with severe PHLF). PHLF, post-hepatectomy liver failure; ECOG, Eastern Cooperative Oncology Group; CCI, Charlson co-morbidity index; HUI, hepatic uptake index (of the unstandardized future liver remnant); sFLR-HUI, hepatic uptake index of the standardized future liver remnant; MELD 3 score, Model for End-Stage Liver Disease Version 3 score; ALBI score, albumin-bilirubin score; FLRV, future liver remnant volume; sFLR, standardized future liver remnant; FLIS, functional liver imaging score.

### Differences in MRI and laboratory parameters

Patients with severe PHLF had lower HUI values (median 331 (interquartile range (i.q.r.) 189–441) *versus* 536 (i.q.r. 351–944); *P* < 0.001), lower sFLR-HUI values (median 18.1 (i.q.r. 10.9–29.4) *versus* 34.0 (i.q.r. 20.8–56.5); *P* < 0.001), a lower FLIS (median 6 (i.q.r. 4.5–6) *versus* 6 (i.q.r. 6–6); *P* = 0.031), and higher MELD 3 scores (median 7.5 (i.q.r. 7–11) *versus* 6 (i.q.r. 6–8); *P* < 0.001). Their sFLR was also significantly smaller (median 36.8% (i.q.r. 26.5%–46.2%) *versus* 41.1% (i.q.r. 32.3%–58.8%); *P* = 0.037). Patients with severe PHLF were less likely to have an sFLR >30% (AR 2.1 for no PHLF) or an sFLR >40% (AR 2.3 for no PHLF) (see *Table 1* for details). Patients with severe PHLF who underwent bile duct resection had a significantly smaller sFLR and significantly lower HUI and sFLR-HUI values, whereas MELD 3 scores did not differ significantly, compared with patients with severe PHLF without bile duct resection (*Table S1*). The general characteristics of patients with and without bile duct resection are detailed in *Table S2*.

### Correlation analysis

There was a moderate negative correlation between the HUI and severe PHLF (Spearman’s ρ = −0.250; *P* < 0.001), indicating that severe PHLF was more common in patients with a lower HUI. The sFLR-HUI was negatively correlated with severe PHLF (Spearman’s ρ = −0.242; *P* < 0.001). MELD 3 scores were positively correlated with severe PHLF (Spearman’s ρ = 0.223; *P* < 0.001). Additionally, background liver disease (Cramér’s V = 0.230; *P* = 0.008), particularly cirrhosis, and type of resection (Cramér’s V = 0.223; *P* = 0.007), specifically extended right hemihepatectomy, as well as bile duct resection (Phi = 0.156), were positively correlated with severe PHLF. The sFLR (Spearman’s ρ) and FLIS showed a weak negative correlation, but the 95% confidence interval included zero. Correlation data are shown in more detail in *Table 1*.

### Variables without significant differences

Other variables, including age, sex, Eastern Cooperative Oncology Group (ECOG) performance status score, ASA grade, BMI, diabetes, Charlson co-morbidity index, type of tumour resected, preoperative volume augmentation, ALBI score, FLRV, and sFLR >20%, did not significantly differ between groups (details in *Table 1*).

### Binary logistic regression

Binary logistic regression analysis assessed the association of the sFLR, sFLR20, sFLR30, sFLR40, HUI, sFLR-HUI, ALBI score, and MELD 3 score with severe PHLF. The HUI, sFLR-HUI, and MELD 3 score had stronger associations with severe PHLF than the sFLR or its derived thresholds, with HUI demonstrating the highest explanatory value based on the Nagelkerke R^2^ (0.162). Highly significant associations were found for the HUI (*P* < 0.001), sFLR-HUI (*P* < 0.001), and MELD 3 score (*P* < 0.001). The sFLR had a weaker association (Nagelkerke R^2^ = 0.040), but remained statistically significant (*P* = 0.041), as did sFLR30 (Nagelkerke R^2^ = 0.031) and sFLR40 (Nagelkerke R^2^ = 0.042). Neither the ALBI score (*P* = 0.102) nor sFLR20 (*P* = 0.111) was significantly associated with severe PHLF. *Table 2* gives more detailed information.

**Table 2:** ORs, both standardized and Z-score standardized, with significance and Nagelkerke R^2^ for the variables HUI, sFLR-HUI, MELD 3 score, ALBI score, sFLR, sFLR20, sFLR30, and sFLR40

Variable	OR (95% c.i.)	Z-score OR (95% c.i.)	*P*	Nagelkerke R^2^
HUI	0.996 (0.994,0.998)	0.116 (0.034,0.388)	<0.001*	0.162
sFLR-HUI†	0.946 (0.916,0.977)	0.136 (0.043,0.432)	<0.001*	0.156
MELD 3 score‡	1.299 (1.145,1.474)	1.888 (1.390,2.566)	<0.001*	0.136
ALBI score§	2.184 (0.856,5.573)	1.425 (0.932,2.181)	0.102	0.029
sFLR†	0.970 (0.943,0.999)	0.571 (0.333,0.977)	0.041*	0.040
sFLR20†	3.848 (0.733,20.211)	1.250 (0.950,1.646)	0.111	0.016
sFLR30†	2.520 (1.043,6.088)	1.456 (1.017,2.084)	0.040*	0.031
sFLR40†	2.790 (1.119,6.954)	1.671 (1.058,2.641)	0.028*	0.042

^*^Statistically significant. †Data missing for eight patients (7 patients with no or mild PHLF and 1 patient with severe PHLF). ‡Data missing for four patients (3 patients with no or mild PHLF and 1 patient with severe PHLF). §Data missing for 116 patients (100 patients with no or mild PHLF and 6 patients with severe PHLF). HUI, hepatic uptake index (of the unstandardized future liver remnant); sFLR-HUI, hepatic uptake index of the standardized future liver remnant; MELD 3 score, Model for End-Stage Liver Disease Version 3 score; ALBI score, albumin-bilirubin score; sFLR, standardized future liver remnant (sFLR20, sFLR threshold at 20% remaining sFLR; sFLR30, sFLR threshold at 30% remaining sFLR; and sFLR40, sFLR threshold at 40% remaining sFLR).

### ROC analysis

**Fig. 2:**
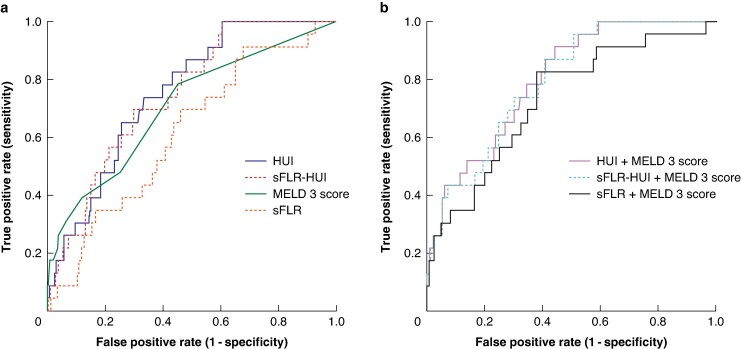
ROC curves comparing discriminatory performance for severe PHLF **a** ROC analysis for the variables HUI, sFLR-HUI, MELD 3 score, and sFLR. AUC: HUI, 0.758; sFLR-HUI, 0.751; MELD 3 score, 0.705; and sFLR, 0.628. **b** ROC analysis for the combinations HUI + MELD 3 score, sFLR-HUI + MELD 3 score, and sFLR + MELD 3 score. AUC: HUI + MELD 3 score, 0.803; sFLR-HUI + MELD 3 score, 0.797; and sFLR + MELD 3 score, 0.733. ROC, receiver operating characteristic; PHLF, post-hepatectomy liver failure; HUI, hepatic uptake index (of the unstandardized future liver remnant); sFLR-HUI, hepatic uptake index of the standardized future liver remnant; MELD 3 score, Model for End-Stage Liver Disease Version 3 score; sFLR, standardized future liver remnant; AUC, area under the curve.

ROC analysis indicated that the HUI had moderate to good predictive ability for severe PHLF (AUC 0.758) and the sFLR-HUI had a similar AUC (0.751). The MELD 3 score was acceptable (AUC 0.705), whereas the sFLR score had a lower AUC of 0.628 (*Fig. 2a*). The FLIS achieved an AUC of 0.605.

At the maximum for the Kolmogorov–Smirnov statistic (model best fit), the HUI reached 75% sensitivity and 66.2% specificity at a value of 415. The sFLR-HUI was comparable, with a lower sensitivity of 70.8%, but a higher specificity of 69.6%, at a value of 23.5. The sFLR reached 70.8% sensitivity and 52.8% specificity at sFLR volumes of 39.8%.

Cut-off values and specificities for 100%, 95%, and 90% sensitivity are also given in *Table 3*.

**Table 3:** Diagnostic accuracy of HUI, sFLR-HUI, and sFLR for severe PHLF

	Sensitivity (%)	Specificity (%)	Cut-off (% for sFLR)
HUI	100	39.2	697
	95	39.2	690
	90	44.2	608
	75*	66.2*	415*
sFLR-HUI	100	39.2	43.2
	95	40.4	41.8
	90	42.3	40.6
	70.8*	69.6*	23.5*
sFLR	100	6.9	79.4
	95	9.2	74.0
	90	31.5	52.0
	70.8*	53.8*	39.8*

Cut-off values are provided for different levels of sensitivity. *Values at the maximum for the Kolmogorov–Smirnov statistic across all possible cut-off points for each test variable. HUI, hepatic uptake index (of the unstandardized future liver remnant); sFLR-HUI, hepatic uptake index of the standardized future liver remnant; sFLR, standardized future liver remnant; PHLF, post-hepatectomy liver failure.

The results of the pairwise comparisons between the different ROC analyses, including *P* values and 95% confidence intervals, are given in (*Table S3*).

ROC analysis for patients undergoing major resection with and without bile duct resection demonstrated a reduction in AUC for the HUI (from 0.758 to 0.685), sFLR-HUI (from 0.751 to 0.668), and sFLR (from 0.628 to 0.517) in patients without bile duct resection. The performance of the MELD 3 score and FLIS (within its subcohort) remained essentially unchanged (AUC changed from 0.705 to 0.703 and from 0.605 to 0.610 respectively).

**Fig. 3:**
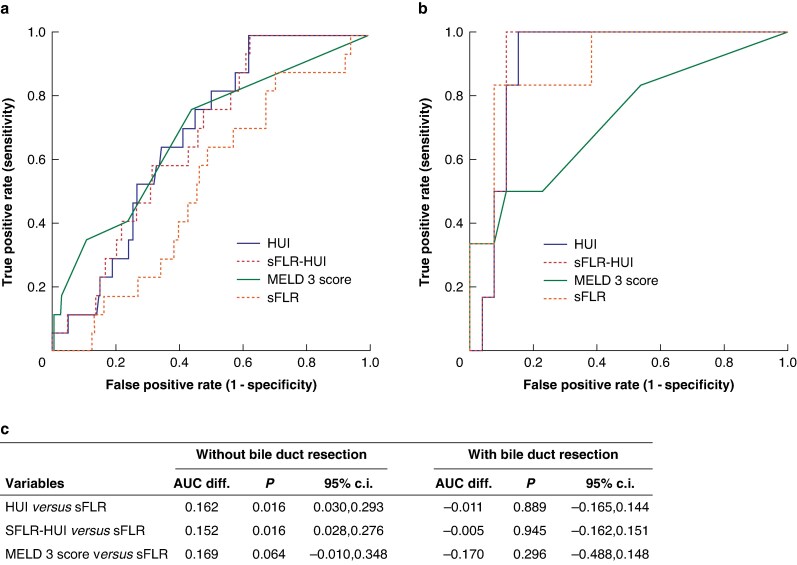
ROC curves for identifying patients at risk of severe PHLF, for patients with and without bile duct resection ROC analysis for the variables HUI, sFLR-HUI, MELD 3 score, and sFLR for identifying patients at risk of severe PHLF. **a** Patients who underwent major resection without bile duct resection. AUC: HUI, 0.685; sFLR-HUI, 0.668; MELD 3 score, 0.703; and sFLR, 0.517. **b** Patients who underwent major resection with bile duct resection. AUC: HUI, 0.884; sFLR-HUI, 0.907; MELD 3 score, 0.713; and sFLR, 0.912. **c** Results of the pairwise AUC comparison of HUI, sFLR-HUI, and MELD 3 score *versus* sFLR for major resections with and without bile duct resection. ROC, receiver operating characteristic; PHLF, post-hepatectomy liver failure; HUI, hepatic uptake index (of the unstandardized future liver remnant); sFLR-HUI, hepatic uptake index of the standardized future liver remnant; MELD 3 score, Model for End-Stage Liver Disease Version 3 score; sFLR, standardized future liver remnant; AUC, area under the curve; diff., difference.

Patients with bile duct resection demonstrated an increase in AUC for the HUI (from 0.758 to 0.884) and sFLR-HUI (from 0.751 to 0.907), with the most pronounced improvement observed for the sFLR (from 0.628 to 0.912). The MELD 3 score and FLIS showed only minor variation (AUC changed from 0.705 to 0.713 and from 0.605 to 0.566 respectively). ROC curves and paired comparison results are presented in *Fig. 3* for the HUI, sFLR-HUI, MELD 3 score, and sFLR. ROC curves for patients with and without bile duct resection that include the FLIS are shown in *Fig. S1*.

### Multivariable evaluation

Combining the MELD 3 score with the HUI, sFLR-HUI, or sFLR yielded higher discriminative performance than any single variable alone. The HUI + MELD 3 score model had the highest AUC (0.803) and reached 91.7% sensitivity at a specificity of 56.1%. This model explained 25.5% of the variance (Nagelkerke R^2^ = 0.255) and there was a statistically significant difference in AUC in the pairwise comparison with the HUI alone (*P* = 0.020 (95% c.i. 0.009 to 0.103)). The performance of the sFLR-HUI + MELD 3 score model was comparable, although slightly lower (AUC 0.797), with 87% sensitivity at a specificity of 59.5%. Compared with the sFLR alone, the combination of the sFLR and the MELD 3 score showed improved discriminative ability (AUC 0.733, 82.6% sensitivity, and 61.9% specificity), but remained inferior to HUI-based models (*Fig. 2b*). However, this difference was not significant in pairwise comparison (HUI + MELD 3 score *versus* sFLR + MELD 3 score: *P* = 0.057 (95% c.i. −0.002 to 0.143)).

## Discussion

The HUI obtained from routine Gd-EOB MRI outperforms standard volumetric indicators, such as the sFLR, in the identification of patients at risk of severe PHLF and can be used for risk stratification of patients before major hepatectomy. The performance of the HUI can be increased when combined with other independent predictors, especially the MELD 3 score. Future efforts should focus on integrating MRI-derived functional assessments with other routine indicators in multivariable models to optimize preoperative risk stratification and outcomes after major liver surgery.

This study evaluates the potential of Gd-EOB MRI for identifying patients at risk of severe PHLF^26^. A total of 292 patients were included, 25 of whom developed severe PHLF (ISGLS grades B and C). Although the number of severe PHLF cases was relatively small, it exceeded that of most other studies, which often include fewer than ten cases^22,27–29^. Consistent with previous research, patients who developed severe PHLF had significantly lower surrogate measurements of liver function in their FLR^20,22,26–28,30^, specifically lower HUI and sFLR-HUI values.

The HUI showed significant discriminative performance for severe PHLF, surpassing purely volume-based indicators, such as the sFLR, and contributed more to model variance in regression analysis. At an HUI cut-off of 415, sensitivity for severe PHLF reached 75% with 66.2% specificity. Adjusting the HUI by BSA (sFLR-HUI) did not improve accuracy, consistent with previous findings^27^. This may be due to minimal BMI and BSA differences across cohorts, including the present study.

Adding the MELD 3 score to the HUI increased performance, reaching 91.7% sensitivity at 56.1% specificity. Patients at very low risk of severe PHLF could be identified at an HUI >697, with none developing severe PHLF, enabling classification of approximately 36% of patients as very low risk.

The diagnostic accuracy of the HUI aligns with previous studies, such as the study by Lauscher *et al*.^22^. However, the discriminative values observed were lower than those reported by Yu *et al*.^27^ and Notake *et al*.^28^. Notake *et al*.^28^ identified a threshold value of 0.41 for predicting severe PHLF with 100% sensitivity and 73% specificity, corresponding to an HUI value of 410 in the present study. While this is close to the identified HUI threshold of 415, specificity was significantly lower, likely due to scanner and protocol heterogeneity in this multicentre study. Although this increases the generalizability of the findings, it also highlights the limitations of relying on routine clinical MRI, especially in a multicentre setting.

The HUI also outperformed the sFLR in identifying patients at risk of severe PHLF, aligning with the findings of Olthof *et al*.^18^. The strongest sFLR performance was noted around sFLR volumes of 40% (AUC 0.628, 70.8% sensitivity, and 53.8% specificity), reinforcing the relevance of the 40% sFLR threshold generally reserved for patients with cirrhosis. However, the sFLR-derived threshold values did not demonstrate strong associations with severe PHLF. While smaller sFLR volumes are linked to increased risk, the sFLR20 threshold failed to show a significant association with severe PHLF in this patient cohort and sFLR30 demonstrated only a weak association. This stresses the limitations of the sFLR, as a substantial proportion of patients undergoing major hepatectomy will have sFLR volumes <40%.

Additionally, the discriminative value of the HUI exceeded that of HBS, as reported by Olthof *et al*.^18^. This may arise because the HUI is not purely volumetric (sFLR) or functional (HBS), but is a combination of both and thus more accurately represents the functional reserve of the FLR.

This is further emphasized when comparing patients with and without bile duct resection. Among patients with severe PHLF, those who underwent bile duct resection had a significantly smaller sFLR than patients without bile duct resection (median sFLR of 25.5% *versus* median sFLR of 38.6%; *P* = 0.003), placing them at higher risk of severe PHLF based on volumetric criteria alone. In this subgroup, the sFLR performed exceptionally well (AUC 0.912), even surpassing the HUI (AUC 0.884) likely due to the dominant effect of severely reduced liver remnant volume, potentially further compounded by complications such as postoperative infections, which were more common in this group. The MELD 3 score did not differ significantly between patients with and without bile duct resection, both in the overall cohort and among those with severe PHLF, suggesting that overall liver function was comparable across groups. In contrast, patients with severe PHLF without bile duct resection typically had larger FLRVs, reducing the relevance of volume, and shifting emphasis toward functional aspects of the FLR. As a result, sFLR accuracy declined to poor (AUC 0.517), whereas the HUI maintained fair performance (AUC 0.685). These findings illustrate the advantage of biomarkers like the HUI, which integrate both regional functional and volumetric components for risk stratification.

Given the multifactorial nature of PHLF, a more comprehensive approach for risk stratification and prediction is needed, as supported by multiple studies^31–35^. As seen in this study, combining the HUI with the MELD 3 score increased the discriminative performance. Santol *et al*.^13^ and Xu *et al*.^36^ demonstrated promising predictive results using multivariable models based on routine tests and intraoperative data, but did not incorporate imaging-derived functional and volumetric assessments of the liver, particularly the FLR. This presents an important opportunity for MRI-derived biomarkers, such as the HUI, which integrate both functional and volumetric aspects of the FLR.

Though this study focused on the HUI, no consensus exists on the most consistent MRI-derived surrogate marker of liver function, as noted by Wang *et al*.^20^. The relative liver enhancement index has shown promise for quantifying liver function in cirrhotic patients^37^ and in primary sclerosing cholangitis^38^. However, it requires measurements from both native and hepatobiliary phases, which can pose challenges in multicentre settings, where imaging protocols may not be standardized. It also lacks volumetric integration, a key factor in PHLF assessment, and, like the HUI, it requires multiple signal intensity measurements that increase the workload and make generalizability across scanners and protocols difficult. In contrast, the FLIS^24,25,39^ offers a semi-quantitative alternative based solely on visual assessment of liver enhancement and bile secretion. It is quicker, easier to apply, and more resistant to scanner and protocol variations, but does not incorporate volume. In this study, the FLIS performed comparably to the sFLR (AUC 0.575 *versus* 0.642; *P* = 0.557), but significantly worse than the HUI (AUC 0.605 *versus* 0.799; *P* = 0.038). While less powerful than the HUI, the FLIS is more robust and easier to implement. Both may play complementary roles in multivariable predictive models for PHLF.

When incorporating MRI-based parameters into the diagnostic process, cost considerations are essential. In this study, the HUI was derived from Gd-EOB MRI examinations already performed as part of routine preoperative workup. In this context, HUI calculations would only add minimal additional work and cost. However, if Gd-EOB MRI was to be adopted as a universal standard before major liver resections, it may impose increased costs, particularly in settings with limited MRI availability. Future research should assess whether MRI-derived biomarkers provide sufficient clinical benefit to justify their cost and whether a targeted, risk-stratified approach would be more practical than broad implementation.

In addition to the heterogeneity introduced by the multicentre approach, this study has several limitations, such as the retrospective design and the relatively small number of patients with severe PHLF. This restricted the number of variables that could be combined without risking overfitting. HUI measurements based on multiple ROIs over the liver and spleen introduced sampling bias^20^, whereas the binary mask-based approach of Lauscher *et al*.^22^ may reduce selection bias. Variability in hepatobiliary phase timing further contributed to heterogeneity. Direct comparisons with established liver function markers, such as ICG clearance or ^99m^Tc-mebrofenin scintigraphy, were not available, but would have strengthened the credibility of the HUI as an alternative or complementary method. The small number of cirrhotic and volume-augmented patients precluded meaningful subgroup analyses. Additionally, assessing the FLIS in only a randomly selected subset of patients may have skewed its evaluation. Finally, for patients with atypical resections, apart from the inclusion criteria, the available data did not allow independent verification that all cases met the definition of major resection.

## Supplementary Material

znaf103_Supplementary_Data

## Data Availability

Data may be available upon reasonable request, but access may be limited due to the conditions stated in the ethics permits for the research performed.
