# Supplementary material for: Magnetic resonance-derived hepatic uptake index improves the identification of patients at risk of severe post-hepatectomy liver failure
Source: Br J Surg. 2025 May 29;112(5):znaf103. doi: 10.1093/bjs/znaf103 (PMC12120443; doi:10.1093/bjs/znaf103)
Supplement: znaf103_Supplementary_Data [file znaf103_supplementary_data.zip › Supplementary_material.docx]

**Title : Magnetic Resonance–Derived Hepatic Uptake Index Im-proves Identification of Patients at Risk of Severe Posthepatectomy Liver Failure**

Authors: Wolf C. Bartholomä^1,2^, Stefan Gilg^3^, Peter Lundberg^2,4^, Peter Noergaard Larsen^5^, Ville Sallinen^6^, Malin Sternby Eilard^7^, Jozef Urdzik^8^, Gert Lindell^9^, Torkel B. Brismar^10^, Eva Fallentin^11^, Ali Ovissi^12^, Andreas Socratous^13^, Tomas Bjerner^1,2^, Sophie Kollbeck^5^, Jens Tellman^4^, Fredrik Holmquist^14^, Nils Dahlström^1,2^, Mischa Woisetschläger^1,2^, Bergthor Björnsson^15^, Ernesto Sparrelid^3^, Per Sandström^15^

^1^ Department of Radiology, and Department of Health, Medicine and Caring Sciences, Linköping University, Linköping, Sweden.

^2^ Center for Medical Image Science and Visualization (CMIV), Linköping University, Linköping, Sweden.

^3^ Division of Surgery and Oncology, Department of Clinical Science, Intervention and Technology, Karolinska Institutet, Karolinska University Hospital, Stockholm, Sweden.

^4^ Department of Radiation Physics, and Department of Health, Medicine and Caring Sciences, Linköping University, Linköping, Sweden.

^5^ Department of Surgical Gastroenterology and Transplantation, Rigshospitalet, Copenhagen University Hospital, Copenhagen, Denmark.

^6^ Department of Gastroenterological Surgery, Helsinki University Hospital and University of Helsinki, Helsinki, Finland.

^7^ Transplant Institute, Institute of Clinical Sciences, Sahlgrenska Academy at University of Gothenburg, Sahlgrenska University Hospital, Gothenburg, Sweden.

^8^ Department of Surgical Sciences, Uppsala University, Uppsala, Sweden.

^9^ Department of Surgery, Skåne University Hospital Comprehensive Cancer Center, Lund University, Lund, Sweden.

^10^ Department of Radiology, Karolinska University Hospital in Huddinge, Huddinge, Stockholm, Sweden.

^11^ Department of Radiology, Rigshospitalet, Copenhagen University Hospital, Copenhagen, Denmark.

^12^ Department of Radiology, University of Helsinki and Helsinki University Hospital, Helsinki, Finland.

^13^ Department of Radiology, Sahlgrenska University Hospital, Gothenburg, Sweden.

^14^ Department of Medical Imaging and Physiology, Skåne University Hospital, Lund University, Lund, Sweden.

^15^ Department of Surgery, and Department of Biomedical and Clinical Sciences, Linköping University, Linköping, Sweden.

**Corresponding author.** Wolf C. Bartholomä, Department of Radiology, Linköping University, Eklyckegatan 11, 58225 Linköping, Sweden. **ORCID ID** 0000-0002-6897-2717

**Supplementary Materials - Index**

| **Supplementary Methods** |  |
| --- | --- |
| None |  |
|  |  |
| **Supplementary Results** |  |
| None |  |
|  |  |
| **Supplementary Appendixes** |  |
| None |  |
|  |  |
| **Supplementary Figures and Tables** |  |
| **Table S1:** Patients with severe PHLF, without and with bile duct resection – Statistics including P- and R- values for HUI, sFLR-HUI, MELD 3, sFLR, bile leaks, and postoperative infection. | *page. 6* |
| **Figure S1:** ROC analysis results for HUI, sFLR-HUI, MELD 3, FLIS and sFLR for patients with major hepatectomy with and without bile duct resection | *page. 7* |
| **Table S2:** General characteristics of patients with and without bile duct resection | *page. 8,9* |
| **Table S3:** Pairwise ROC comparisons of HUI, sFLR-HUI, MELD 3, HUI + MELD 3, sFLR-HUI + MELD 3, and sFLR + MELD 3 against sFLR, as well as HUI + MELD 3 versus sFLR + MELD 3. | *page. 10* |
|  |  |
|  |  |
|  |  |

**Supplementary Methods**

None.

**Supplementary Results**

None

**Supplementary Appendixes**

None.

**Supplementary Figures and Tables**

**Table S1**: Patients with severe PHLF, without and with bile duct resection – Statistics including P- and R- values for HUI, sFLR-HUI, MELD 3, sFLR, bile leaks, and postoperative infection.

|  | No Bile Duct Resection (n=18) | Bile Duct Resection (n=7) | *P* | *R* |
| --- | --- | --- | --- | --- |
| HUI, median (i.q.r.) | 351 (287 – 493) | 173 (104 – 214) | 0,001^E^* | -0.618^++^ (-0.818 - -0.283) |
| sFLR-HUI^1^, median (i.q.r.) | 23.3 (16.5 – 34.1) | 10.4 (5.6 – 14.5) | 0.001^E^* | -0.642^++^ (-0.834 - -0.311) |
| MELD 3^2^, median (i.q.r.) | 7 (6.75 – 10.25) | 10 (6.75 – 21.5) | 0.251^E^ |  |
| sFLR, median (i.q.r.)^1^ | 38.6 (34.6 – 49.9) | 25.5 (19.0 – 29.0) | 0.003^E^* | -0.589^++^ (-0.807 - -0.232) |
| Bile leak, n(%) | 5 (27.8) | 3 (42.9%) | 0.640^F^ |  |
| Infection, n(%) | 6 (33.3) | 6 (85.7) | 0.030^F^* | 0.471^+^ (0.123 – 0.775) |

^E^ *P* was calculated using Mann-Whitney Exact Test.

^F^ *P* was calculated using Fisher’s Exact Test.

R was calculated using Spearman’s Rho for HUI and sFLR and Phi for Infection. Correlations are given as R (CI). CI Intervals were calculated with Bootstrapping (1000 samples).

^++^ signifies correlation at the 0.01 level.

^+^ signifies correlation at the 0.05 level.

HUI = Hepatic Uptake Index of the unstandardised FLR; sFLR-HUI = Hepatic Uptake Index of the standardised FLR; MELD 3 = Model for End-Stage Liver Disease Version 3; sFLR = standardised Future Liver Remnant

Missing Data:

^1^ 1 patient missing for severe PHLF without bile duct resection.

^2^ 1 patient missing for severe PHLF with bile duct resection.

## Figure S1:

***Fig. S1*** *ROC analysis for the variables HUI, sFLR-HUI, MELD 3, FLIS, and sFLR for identifying patients at risk for severe PHLF.* ***a)*** *Patients who underwent major resection without bile duct resection;* ***b)*** *Patients who underwent major resection with bile duct resection.*

*This is a randomly selected subcohort of the study cohort, consisting of 155 patients, 142 with mild or no PHLF (91.6%), 13 (8.4%) with severe PHLF. For patients with severe PHLF and bile duct resection, only 3 FLIS score evaluations were available.*

*Areas under the curve:* ***a)*** *HUI 0.767, sFLR-HUI 0.718, MELD 3 0.794, FLIS 0.610, sFLR 0.523;* ***b)*** *HUI 0.867, sFLR-HUI 0.889, MELD 3 0.633, FLIS 0.556, sFLR 0.956.*

*HUI = Hepatic Uptake Index of the unstandardised FLR; sFLR-HUI = Hepatic Uptake Index of the standardised FLR; MELD 3 = Model for End-Stage Liver Disease Version 3; sFLR = standardised future liver remnant; FLIS = Functional liver imaging score, FLR = Future liver remnant.*


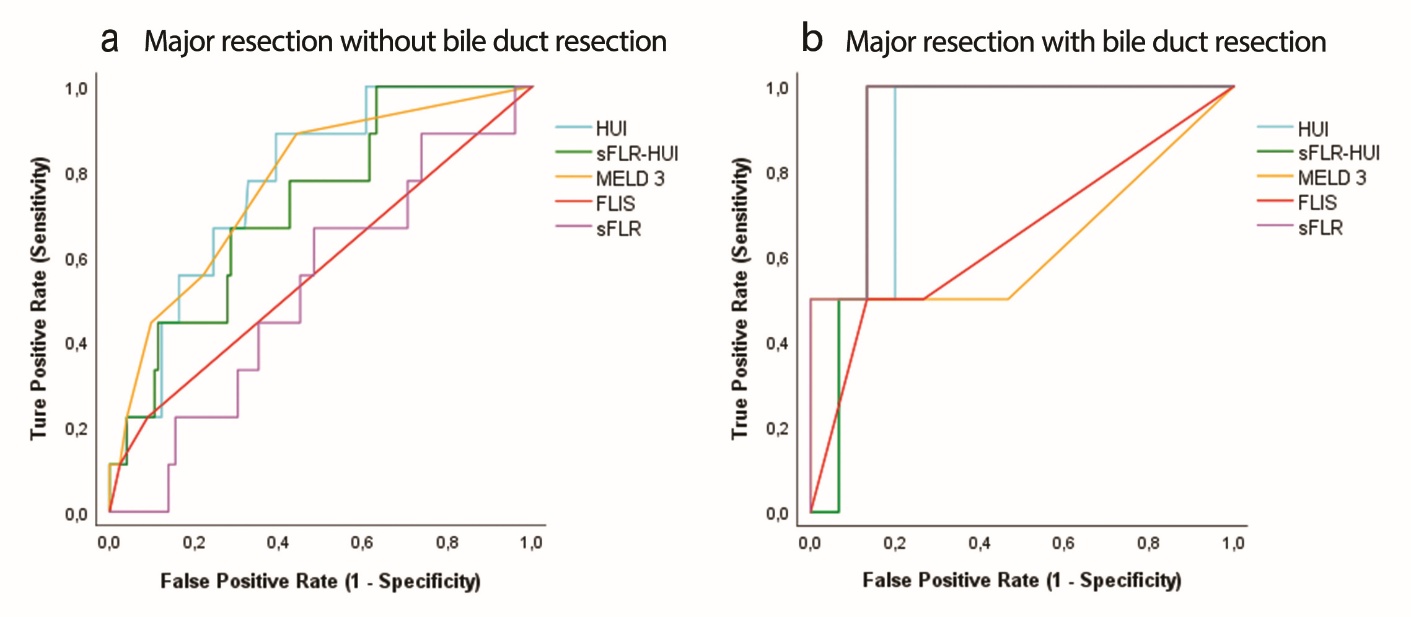


#### Alt Text Figure S1:

Figure showing ROC curves for predicting severe PHLF using various biomarkers in patients with and without bile duct resection, based on a subcohort of 155 patients.
Panel (a): For patients without bile duct resection, HUI (AUC 0.767), sFLR-HUI (0.718), MELD 3 (0.794), FLIS (0.610), and sFLR (0.523).
Panel (b): For patients with bile duct resection, HUI (AUC 0.867), sFLR-HUI (0.889), MELD 3 (0.633), FLIS (0.556), and sFLR (0.956).
FLIS data were limited in the bile duct resection group (only 3 cases).

**Table S2:** Characteristics of patients with and without bile duct resection.

|  | **Total Study population (n=292)** |  |  |  |
| --- | --- | --- | --- | --- |
|  |  |  |  |  |
|  | **No Bile Duct Resection** | **Bile Duct Resection** | ***P*** | ***R*** |
|  |  |  |  |  |
| Total Number of patients per group | 258 (88.4) | 34 (8.6) |  |  |
| **Patient characteristics** |  |  |  |  |
| Age at Surgery (years), median (i.q.r.) | 64 (56-71) | 67 (53-73) | 0.639 |  |
| Sex ratio (M:F) | 159:99 | 21:13 | 0.988 |  |
| ECOG, median (i.q.r.)^4^ | 0 (0) | 0 (0-1) | 0.442 |  |
| ASA, median (i.q.r.)^5^ | 2 (2-3) | 2 (2-3) | 0.716 |  |
| BMI, median (i.q.r.) | 25.86 (23.2-28.8) | 24.3 (22.5-27.4) | 0.088 |  |
| Patient with diabetes mellitus | 35 (13.1) | 4 (16.0) | 0.434 |  |
| **Background Liver Disease** |  |  | 0.553 |  |
| Normal | 71 (31.1) | 10 (33.3) |  |  |
| Steatosis | 46 (20.2) | 3 (10.0) |  |  |
| Fibrosis Grade 1-3 | 86 (37.7) | 15 (50.0) |  |  |
| Cirrhosis | 10 (4.4) | 1 (3.3) |  |  |
| Other | 15 (6.6) | 1 (3.3) |  |  |
| **Type of resected tumour (Histology)** |  |  | <0.001* | 0.590 |
| Colorectal metastases | 169(65.5) | 3 (8.8) |  |  |
| Hepatocellular Cancer | 14 (5.4) | 2 (5.9) |  |  |
| Biliary Cancer (intrahepatic, perihilar, extrahepatic) | 37 (14.3) | 8 (23.5) |  |  |
| Gallbladder Cancer | 3 (1.2) | 14 (41.2) |  |  |
| Other (benign, other malign, non specified) | 35 (13.6) | 7 (20.6) |  |  |
| **Type of Resection** |  |  | <0.001* | 0.427 |
| Right Hemihepatectomy | 161 (62.6) | 4 (13.8) |  |  |
| Extended Right Hemihepatectomy | 26 (10.1) | 12 (41.4) |  |  |
| Left Hemihepatectomy | 40 (15.6) | 7 (24.1) |  |  |
| Extended Left Hemihepatectomy | 7 (2.7) | 6 (20.7) |  |  |
| Atypical Resection | 23 (8.9) | 0 (0.0) |  |  |
| **Severe PHLF** |  |  | 0.008* | 0.156^+^ |
| PHLF Grade B+C | 18 (7.0) | 7 (20.6) |  |  |
| **Parameters** |  |  |  |  |
| HUI, median (i.q.r.) | 501 (335-884) | 570 (198-1133) | 0.792 |  |
| sFLR-HUI, median (i.q.r.)^6^ | 31.0 (20.0-51.1) | 37 (13.5-73.8) | 0.961 |  |
| MELD Score (MELD 3), median (i.q.r.)^7^ | 6 (6-8) | 7 (6-9.5) | 0.016* | 0.143^+^ |
| ALBI Score, median (i.q.r)^8^ | -2.49 (-2.73- -2.27) | -2.37 (-2.63- -1.55) | 0.009* | 0.193^++^ |
| FLRV (mL), median (i.q.r.) | 667 (5180-921) | 785 (508-1195) | 0.164 |  |
| sFLR(%), median (i.q.r.)^6^ | 39.7 (31.8-55.2) | 47.2 (30.0-71.9) | 0.138 |  |

Values are *n (%)* unless otherwise indicated. Statistically significant differences are marked with *. R = Spearman’s ρ for continuous data or Phi and Cramér’s V for categorical data. Correlations are only given for variables with statistically significant differences. ^++^ marks correlation at the 0.001 level, ^+^ marks correlation at the 0.05 level.

ECOG = Eastern Cooperative Oncology Group Performance Status; ASA = American Society of Anesthesiologists Physical Status Classification; BMI = Body Mass Index; HUI = Hepatic Uptake Index of the unstandardised FLR; sFLR-HUI = Hepatic Uptake Index of the standardised FLR; MELD 3 = Model for End-Stage Liver Disease Version 3; ALBI Score = Albumin-Bilirubin Score; FLRV = Future Liver Remnant Volume; sFLR = standardised Future Liver Remnant

Missing data:

^1^ Histological Data was missing in 26 of 222 patients without PHLF. Exact information of the type of major resection was missing in 4 of 222 patients.

^2^ Histological Data was missing in 5 of 45 patients with mild PHLF (ISGLS Grade A).

^3^ Histological Data was missing in 3 of 25 patients with PHLF ISGLS Grade B and C. Exact information of the type of major resection was missing in 2 of 25 patients.

^4^ Data is missing for 54 patients (48 patients without BD Resection, 6 patients with BD Resection).

^5^ Data is missing for 2 patients (1 patient without BD resection, 1 patient with BD resection).

^6^ Data is missing for 8 patients (7 patients without BD resection, 1 patient with BD resection).

^7^ Data is missing for 4 patients (3 patients without BD resection, 1 patient with BD resection).

^8^ Data is missing for 116 patients (102 patients without BD resection, 14 patients with BD resection).

**Table S3** Pairwise ROC comparisons of HUI, sFLR-HUI, MELD 3, HUI + MELD 3, sFLR-HUI + MELD 3, and sFLR + MELD 3 against sFLR, as well as HUI + MELD 3 versus sFLR + MELD 3.

| **Comparison** | **AUC (First Variable)** | **AUC (Second Variable)** | ***P*** | **95% CI (Lower-Upper)** |
| --- | --- | --- | --- | --- |
| HUI vs. sFLR | 0.758 | 0.628 | 0.015* | 0.025-0.234 |
| sFLR-HUI vs. sFLR | 0.751 | 0.628 | 0.016* | 0.023-0.222 |
| MELD 3 vs. sFLR | 0.705 | 0.613 | 0.255 | -0.067-0.251 |
| HUI+MELD 3 vs sFLR | 0.803 | 0.613 | 0.001* | 0.081-0.299 |
| sFLR-HUI+MELD 3 vs sFLR | 0.797 | 0.613 | 0.001* | 0.080-0.287 |
| sFLR+MELD 3 vs sFLR | 0.733 | 0.613 | 0.019* | 0.019-0.220 |
| HUI+MELD 3 vs sFLR+MELD 3 | 0.803 | 0.733 | 0.057 | -0.002-0.143 |

HUI = Hepatic Uptake Index of the unstandardised FLR; sFLR = standardised Future Liver Remnant; sFLR-HUI = Hepatic Uptake Index of the standardised FLR; MELD 3 = Model for End-Stage Liver Disease Version 3; AUC = Area under the curve; CI = Confidence Interval

P – Values and 95% CI were calculated in SPSS using the Delong method for pairwise comparison of the Area Under the Curve (AUC) in Receiver Operating Characteristic (ROC) analysis.

Statistically significant differences are marked with *.

**References**

None.
